# Insight into Conformational Change for 14-3-3σ Protein by Molecular Dynamics Simulation

**DOI:** 10.3390/ijms15022794

**Published:** 2014-02-18

**Authors:** Guodong Hu, Haiyan Li, Jing-Yuan Liu, Jihua Wang

**Affiliations:** 1 Shandong Provincial Key Laboratory of Functional Macromolecular Biophysics, College of Physics and Electronic Information, Dezhou University, Dezhou 253023, China; E-Mails: xzszhgd@163.com (G.H.); tianwaifeixian78@163.com (H.L.); 2 The State Key Laboratory of Bioelectronics, Southeast University, Nanjing 210096, China; 3 Department of Computer and Information Science, Indiana University-Purdue University Indianapolis, Indianapolis, IN 46202, USA; 4 Department of Pharmacology and Toxicology, Indiana University School of Medicine, Indianapolis, IN 46202, USA

**Keywords:** conformational change, 14-3-3σ protein, molecular dynamics simulation

## Abstract

14-3-3σ is a member of a highly conserved family of 14-3-3 proteins that has a double-edged sword role in human cancers. Former reports have indicated that the 14-3-3 protein may be in an open or closed state. In this work, we found that the apo-14-3-3σ is in an open state compared with the phosphopeptide bound 14-3-3σ complex which is in a more closed state based on our 80 ns molecular dynamics (MD) simulations. The interaction between the two monomers of 14-3-3σ in the open state is the same as that in the closed state. In both open and closed states, helices A to D, which are involved in dimerization, are stable. However, large differences are found in helices E and F. The hydrophobic contacts and hydrogen bonds between helices E and G in apo-14-3-3σ are different from those in the bound 14-3-3σ complex. The restrained and the mutated (Arg56 or Arg129 to alanine) MD simulations indicate that the conformation of four residues (Lys49, Arg56, Arg129 and Tyr130) may play an important role to keep the 14-3-3σ protein in an open or closed state. These results would be useful to evaluate the 14-3-3σ protein structure-function relationship.

## 1. Introduction

14-3-3 proteins are a family of highly conserved, acidic proteins ubiquitously expressed in all eukaryotic cells [1]. This family of proteins consists of seven distinct isoforms in human cells (β, ∈, γ, η, σ, τ and ζ) as well as a variety of post-translationally modified forms [2–4]. 14-3-3 proteins act as adaptor proteins that control functions of its target proteins through highly regulated protein-protein interactions. 14-3-3 proteins have been shown to interact with several hundred proteins, some of which play important roles in human diseases and in the life of the cell [5]; the σ isoform, for example, has been implicated in breast cancer [6] and is necessary for proper G_2_ checkpoint function [7]. Depending on the nature of its target protein, 14-3-3 proteins are involved in the regulation and coordination of many cellular processes including cell cycle progression, apoptosis, metabolism, transcriptional regulation of gene expression, and the DNA damage response [8,9].

The crystallographic structures of all seven 14-3-3 isoforms are now known [10] and have revealed that 14-3-3 proteins have characteristic cup-like shape functional dimers with each of the monomers consisting of nine antiparallel α-helices displaying a so-called amphipathic groove [10,11]. All 14-3-3 proteins can form homodimers and heterodimers except the sigma isoform [12,13]. Each monomer can independently bind to a target protein in the binding groove [5] for different motifs. These motifs include the prototype sequence, RSxpS/TxP (mode 1), RxxxpS/TxP (mode 2) and pS/TxCOOH (mode 3), where x stands for any amino acid [14–16]. It seems that the 14-3-3 protein may have a rigid conformation because the conformation of most crystallographic structures for 14-3-3 isoforms are similar in both apo- and ligand-bound forms with a closed state except apo-14-3-3β (PDB ID: 2BQ0) which has been found to have one monomer in an open state while the other is in a closed state [10]. However, the 14-3-3 proteins can bind several hundred diverse target proteins [17,18], and a study by Liu *et al*. has suggested that 14-3-3 proteins may bind to target proteins through the transition between open and closed conformations [19,20].

**Figure 1.**
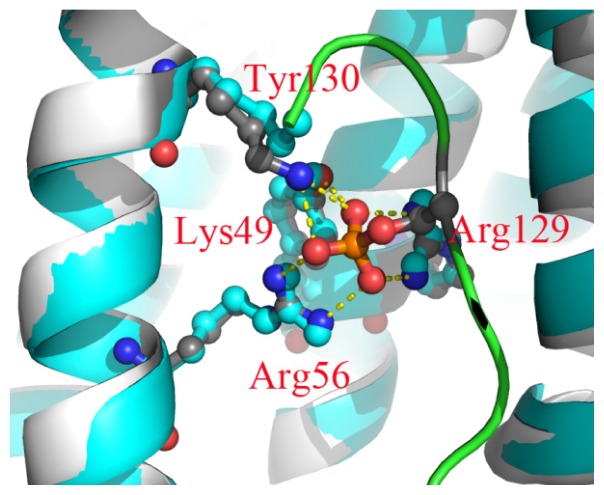
Superimposed structures of 1YWT (white) and 1YZ5 (cyan). The proteins are shown in cartoon representation, and the four residues and the phosphoserine are shown in ball and stick representations.

The crystallographic structure (PDB ID: 1YWT) [12] reveals that the phosphorylated group of the binding peptide formed six hydrogen bonds with 14-3-3σ protein (Figure 1). The six hydrogen bonds involve four hydrophilic residues (Lys49, Arg56, Arg129 and Tyr130). The crystallographic structure of 14-3-3σ protein (PDB ID: 1YZ5) [21] without binding peptide superimposes very well with the 1YWT structure in the closed state. The conformation of the four residues of 1YWT is very similar to 1YZ5 (Figure 1). As we know, the four residues are hydrophilic and would not be stable together in the water environment. These reports and the crystallographic structures raise a series of interesting questions: (1) Is the interaction between the two monomers for the closed state different from that for the open state? (2) Which part of the monomer for the closed state is different from that for the open state? (3) Does the conformation of the four residues interacting with the phosphorylated group influence the state of 14-3-3σ protein? To answer these questions, we utilize MD simulations to analyze the conformational change of 14-3-3σ protein, since MD simulation is an efficient tool for exploring conformational changes and unfolding proteins [22–26].

Here, we will discuss the equilibrium of the MD simulation, the interaction between the two monomers for apo-14-3-3σ and bound 14-3-3σ, the stability for all the helices in 14-3-3σ protein and the reason for the conformational change to understand the differences of 14-3-3σ between closed and open states.

## 2. Results and Discussion

### 2.1. Equilibrium of the Molecular Dynamics Simulation

**Figure 2.**
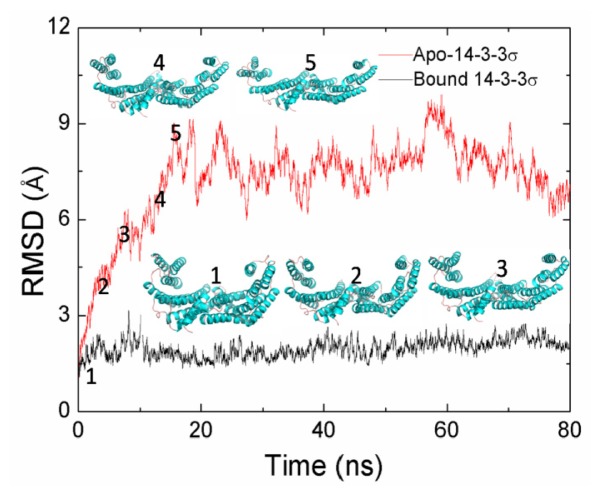
RMSDs of backbone atoms of 14-3-3 protein as a function of the MD simulation time. Five snapshots extracted from the first 20 ns MD simulation of apo-14-3-3σ.

To assess the dynamics stability of the two systems during the MD simulations, energetic and structural properties are monitored during the 80 ns MD simulation for both complexes. Figure 2 shows the root mean square deviations (RMSDs) of the backbone atoms from the starting structures during the MD simulations. As can be seen in Figure 2, the RMSD values of each system tend to equilibrium after 20 ns. The RMSD values for apo-14-3-3σ are larger than those for bound 14-3-3σ during all the MD simulation time, and the averaged RMSD values for the last 60 ns MD simulation trajectories are 7.70 and 2.00 Å for apo-14-3-3σ and bound 14-3-3σ, respectively. This implies that there is large conformational difference between apo-14-3-3σ and bound 14-3-3σ during the MD simulation process. A 60 ns MD simulation for apo-14-3-3σ was carried out with different original speed. It has shown the similar dynamic characters as the first one. So we discuss the conformational change of apo-14-3-3σ according to the first MD simulation.

The 14-3-3 protein dimeric molecule has a characteristic cup-like shape with a central channel, and each monomer of 14-3-3 protein binds to the target protein by the amphipathic ligand-binding groove formed by helices C, E, G and I [27]. In order to understand the influence caused by the absence of the peptide in apo-14-3-3σ, comparisons between the conformation of apo-14-3-3σ and bound 14-3-3σ were performed to obtain global information about the conformational change in the apo-14-3-3σ structure. The last structure of bound 14-3-3σ from the MD trajectories is superimposed with the apo-14-3-3σ structure, as shown in Figure S1. For helices A, B, C and D, which are located on the bottom of each monomer, the structures are superimposed very well for both monomers. However, helices G, H and I, which are located on the top of each monomer in apo-14-3-3σ, are obviously different from those in bound 14-3-3σ. For helices E and F, which are located at the junction between the top and bottom of each monomer, the conformational change is not as large as the helices on the top. As shown in Figure S1, the conformational change can be easily detected by the distances between the two mass centers of helices G, H and I of the two monomers. Figure S2a plots the distances along the 80 ns MD simulation period for apo-14-3-3σ and bound 14-3-3σ. In order to evaluate the dynamic flexibility of helices G, H and I, the linear regressions in apo-14-3-3σ and bound 14-3-3σ are calculated for the last 60 ns and the entire MD simulation time. The distances in bound 14-3-3σ fluctuate around 55.74 Å, as indicated by the slopes of the linear regression lines of −7.05 × 10^−7^ Å/ps. The distances in apo-14-3-3σ are increasing for the first 20 ns MD, and then they tend to fluctuate around 76.53 Å, as indicated by the slopes of the linear regression lines of −8.52 × 10^−6^ Å/ps. Figure S2b and c show the distances between the mass center of helices A to D in the two monomers and the mass center of the helices G to I as a function of MD simulation time. The last structures in apo-14-3-3σ and bound 14-3-3σ are in the closed and the open states, respectively (Figure S1). The distances are less than 35 Å in bound 14-3-3σ (Figure S2b) with the closed state, as well as larger than 37 Å in apo-14-3-3σ after the first 20 ns MD simulation (Figure S2c) with the open state. The distances between the mass center of helices A to D in the both monomers and the mass center of helices G, H and I can be used to define the conformation state of 14-3-3σ.

**Figure 3.**
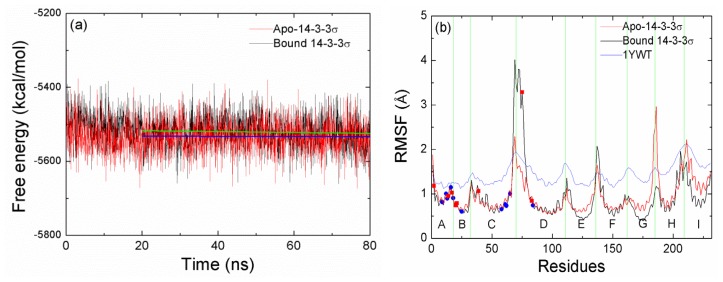
(**a**) Sum of gas-phase and solvation free energies calculated for 2000 snapshots of 14-3-3σ protein extracted at 40 ps intervals from the MD simulations. The linear lines for the last 60 ns simulation time are colored in green and blue for apo-14-3-3σ and bound 14-3-3σ, respectively; (**b**) Root-mean-square fluctuation (RMSF) of the Cα atoms *versus* residue number for a monomer of 14-3-3σ obtained from the crystallographic structure and the last 60 ns MD simulation. The region of helices are divided with the green vertical line and labeled.

In order to complement structural analysis, free energy calculations have been performed by using the MM-GBSA method. Figure 3a shows the time-series of the sums of gas-phase energies and solvation free energies for 14-3-3σ protein without the phosphopeptide, which are computed from 2000 snapshots extracted at 40 ps intervals from the entire MD simulations trajectories. For both cases, the free energies decrease at the original 20 ns MD simulation. After that, the systems tend to reach a stable state, the slopes of linear regression lines for the snapshots from the last 60 ns are −1.23 × 10^−4^ and −2.19 × 10^−5^ kcal/(mol.ps) for apo-14-3-3σ and bound 14-3-3σ, respectively. The enthalpic contribution is averaged over 1500 snapshots taken at 40 ps intervals from the last 60 ns of the MD simulations. The entropic contribution is averaged over 300 snapshots taken at 200 ps intervals from the last 60 ns. As shown in Table 1, the total free energy of apo-14-3-3σ is lower than that of bound 14-3-3σ, this implies that the open conformation of 14-3-3σ protein is more stable than the closed conformation. It means that peptide binding drives 14-3-3 protein transits from open to closed conformation. The total free energy of apo-14-3-3σ protein is unfavorable for the conformational change.

The analysis for the RMSD of backbone atoms indicates that the conformational change of apo-14-3-3σ is large. In order to investigate the stability of the nine helices composing the monomer of 14-3-3 protein, the defined secondary structure of protein (DSSP) [28], which is commonly used to observe the time evolution of protein’s secondary structures, is calculated. Figure S3 plots the DSSP along the 80 ns simulation period. There is no large differences for all the helices between apo-14-3-3σ and bound 14-3-3σ, and the helical structures are stable. However, larger differences are observed at the turn section, which is the junction between two helices, especially for the junction between helices G and H. This implies that the secondary structure of the nine helices for apo-14-3-3σ and bound 14-3-3σ is stable, the large change of apo-14-3-3σ is mainly caused at the junction between the two helices.

Figure 3b illustrates the root mean square fluctuation (RMSF) of the Cα atoms *versus* the residue number for crystallographic structure, apo-14-3-3σ and bound 14-3-3σ, respectively. As seen in Figure 3b, the line for crystallographic structure shows the same character as that for apo-14-3-3σ or bound 14-3-3σ. The RMSF values of the residues in the junction between the two helices are larger than those of the residues in the helices. Comparisons between the RMSF values for every helices in apo-14-3-3σ and bound 14-3-3σ are carried out. It is noteworthy that all the helices show the similar character for both systems except three helices (E, G and I). This indicates that the stabilities for the three helices are larger in bound 14-3-3σ than in apo-14-3-3σ. The crystallographic structure has revealed that these three helices interact with the target protein. Based on these observations, we may draw a conclusion that the bound 14-3-3σ is more rigid than apo-14-3-3σ.

### 2.2. The Interaction between the Two Monomers for Apo-14-3-3σ and Bound 14-3-3σ

**Figure 4.**
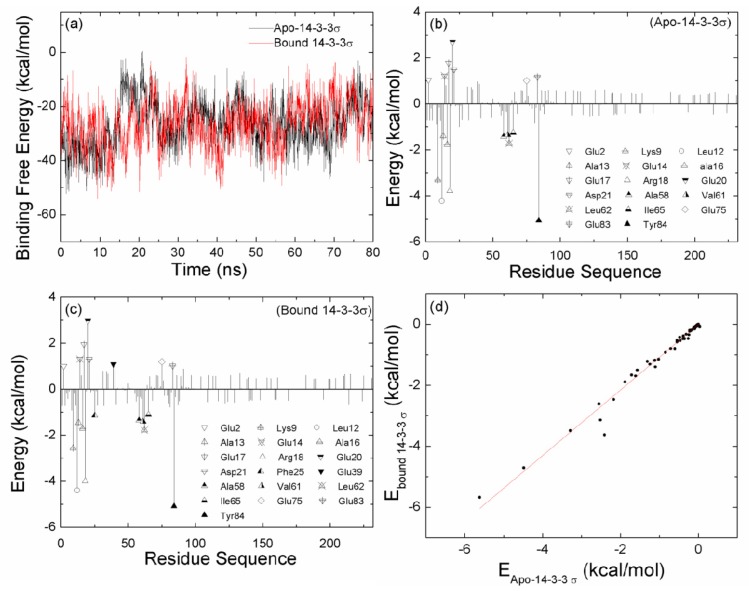
(**a**) Binding free energies calculated for 2000 snapshots extracted at 40 ps intervals from the whole MD simulations for apo-14-3-3σ and bound 14-3-3σ; (**b**) Decomposition of energy on a per-residue basis into contributions from van der Waals energy (*E*_vdW_), sum of electrostatic energies and polar component of solvation free energy (*E*_ele_ + *G*_pol_) and the non-polar (*G*_nonpol_) component of solvation free energy for residues of apo-14-3-3σ, as well as (**c**) for bound 14-3-3σ; (**d**) Comparison between the hydrophobic interactions for per residue in apo-14-3-3σ and bound 14-3-3σ.

As shown in Figures 2 and S2, the conformation for apo-14-3-3σ continues to change and does not reach a stable state during the first 20 ns MD simulation. In order to investigate the interaction between the two monomers for apo-14-3-3σ and bound 14-3-3σ, the binding free energies between the two monomers are calculated with the MM-GBSA method. The binding free energies large fluctuation would indicate a large change for the interaction. Figure 4a shows the comparison of time evolution of the binding free energies between the two monomers in the apo-14-3-3σ and the bound 14-3-3σ. As shown in Figure 4a, the large fluctuation binding free energies for apo-14-3-3σ is observed for the first 20 ns MD simulation; the binding free energies are stable for the both systems after the first 20 ns MD simulation. This is in accordance with the conformational analysis. The averaged binding free energies from the last 60 ns MD simulation are −25.40 and −25.21 kcal/mol for apo-14-3-3σ and bound 14-3-3σ, respectively. This implies that the dimerization affinity for apo-14-3-3σ is the same as the affinity for bound 14-3-3σ.

In order to further explore the detailed interaction, the binding free energies between the two monomers are decomposed on a per residue basis by using the MM-GBSA approach for generating a protein-residue interaction spectrum which is shown in Figure 4b,c. The decomposition approach is extremely useful to locate the residues, which contribute to the protein-protein and protein-ligand interactions [29–31]. The residues with the absolute free energies contribution larger than 1 kcal/mol for the protein-residue are labeled (Figure 4b,c). In this work, comparisons between the interaction spectrums of apo-14-3-3σ and bound 14-3-3σ are performed. The number of the favorable residues for dimerization is eleven and ten in bound 14-3-3σ and apo-14-3-3σ, respectively. And the number of the unfavorable residues for dimerization is eight and seven in bound 14-3-3σ and apo-14-3-3σ, respectively. The residues with large contribution in apo-14-3-3σ are also found in bound 14-3-3σ with large contribution. The interaction spectrum of apo-14-3-3σ is very similar with that of bound 14-3-3σ.

Liu *et al*. have reported that the structural stability of hydrophobic cores is critical for the stable 14-3-3 dimeric protein [19]. The contribution of the hydrophobic interaction (the sum of the van der Waals energy and the non-polar contribution to solvation free energy) for per residue in apo-14-3-3σ are compared with those in bound 14-3-3σ and shown in Figure 4d. The correlation coefficient *r =* 0.99 is obtained; this implies that the hydrophobic contribution for apo-14-3-3σ is the same as for bound 14-3-3σ. We further determine the structural stability of the residues which have large contribution for the binding by calculating the RMSF. The residues unfavorable for binding are in square symbol and red color, and the residues favorable for binding are in circle symbol and blue color (Figure 3b). As shown in Figure 3b, all the RMSF values except one residue Glu75 in bound 14-3-3σ are not larger than those in apo-14-3-3σ. This indicates that the binding pocket of apo-14-3-3σ or bound 14-3-3σ is stable, the change absent peptide in apo-14-3-3σ does not cause change for the interaction between the two monomers.

### 2.3. The Stability of the Helices A, B, C and D

**Figure 5.**
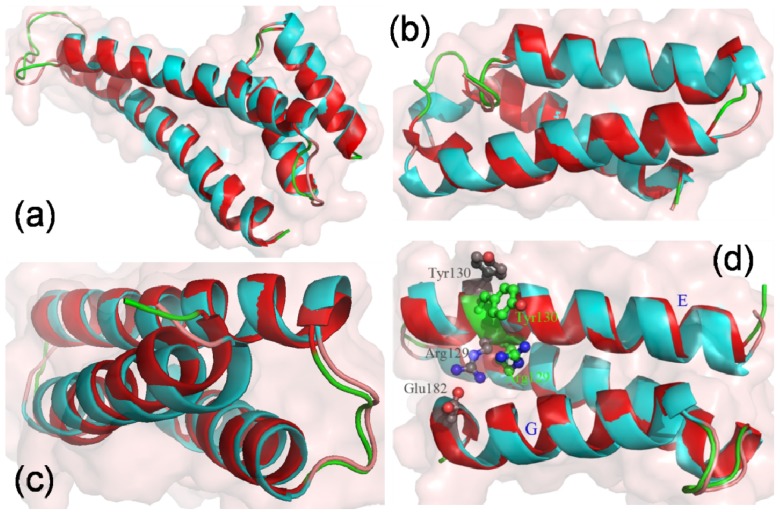
The helices of apo-14-3-3σ (colored in cyan) are superimposed with the helices of bond 14-3-3σ (colored in red) structure. (**a**) is for the helices A, B, C and D; (**b**) is for the helices G, H and I; (**c**) is for the helices E, F and G; (**d**) is for the helices E, F and G in side view with several labeled residues.

Main-chain-based clustering analysis is also performed to investigate conformational fluctuation and stability for helices A, B, C and D. There are six heavy atoms clusters for bound 14-3-3σ, which is in the closed state, as well as 27 for apo-14-3-3σ in the open state. The same cluster analysis is also performed for helices A, B, C and D; there are 5 clusters for both bound 14-3-3σ and apo-14-3-3σ. One of the five clusters is highly populated with a percentage of all the structure 49.9% and 49.8% for bound 14-3-3σ and apo-14-3-3σ, respectively. The structure which is the nearest cluster centre of the highly populated cluster for bound 14-3-3σ is superimposed with that for apo-14-3-3σ and shown in Figure 5a. As shown in Figure 5a, the structure of helices A, B, C and D in bound 14-3-3σ is superimposed very well with that in apo-14-3-3σ. The RMSD value of the backbone for the two nearest cluster centre of the highest populated cluster is 1.96 Å. This implies that the structural changes of these four helices are smaller than the others in apo-14-3-3σ. It is plotted for the RMSD of the backbone atoms of the helices A, B, C and D *versus* the MD simulation times shown in Figure S4a. Comparisons of RMSDs for the whole protein (Figure 2) and for four helices were performed. The RMSDs of apo-14-3-3σ are higher than those of bound 14-3-3σ, however, this difference is obviously smaller than that shown in Figure 2.

### 2.4. The Stability of Helices G, H and I

Helices G, H and I of apo-14-3-3σ are obviously different from the helices in bound 14-3-3σ. In order to investigate the conformational change and stability of these three helices, cluster analysis of heavy atoms was performed. There are six heavy atom clusters for the helices G, H and I for bound 14-3-3σ, as well as five clusters for apo-14-3-3σ. The highest populated clusters are chosen from the clusters for bound 14-3-3σ and apo-14-3-3σ. The structure, with the smallest RMSD from the averaged structure in the highest populated cluster for bound 14-3-3σ is superimposed with that in apo-14-3-3σ and shown in Figure 5b. The two structures superimposed very well. This implies that the stability of the three helices in apo-14-3-3σ is very similar to that in bound 14-3-3σ. The RMSDs of the backbone atoms of the helices G, H and I *versus* the MD times were plotted in Figure S4b.

### 2.5. The Conformational Change of the Helices E, F and G

Figure S5a shows the RMSDs of the backbone atoms for the helices E, F and G from the starting structures during the MD simulation. The RMSDs of the helices E, F and G in apo-14-3-3σ are larger than those in bound 14-3-3σ. The structures which are the nearest to the cluster centers for the populated clusters of the helices E, F and G for apo-14-3-3σ and for bound 14-3-3σ are superimposed and shown in Figure 5c. It is obviously shown in Figure 5c that there are large differences for the relative position of the helices E and G. The distances between the mass center of the helix E and G are calculated during the MD simulation and shown in Figure S5b. The distances and the fluctuation of distance in apo-14-3-3σ are larger than those in bound 14-3-3σ except during first five ns MD simulation. It is quite obvious that the structures of the helices E and G in bound 14-3-3σ are different from those in apo-14-3-3σ.

**Figure 6.**
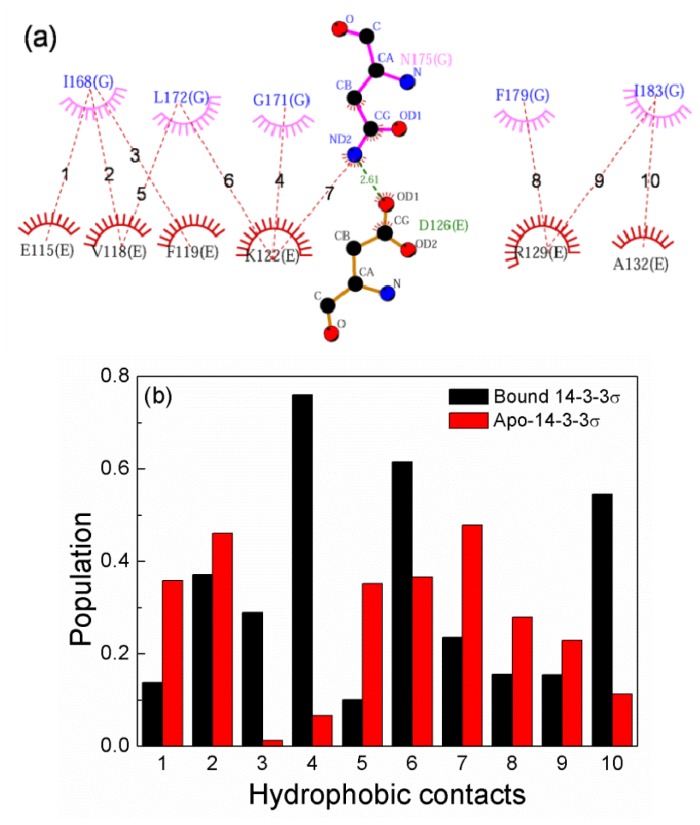
(**a**) Two-dimensional representation for the interactions between helices E and G, the hydrophobic contacts and hydrogen bond are marked with red and green dash line, respectively; (**b**) The populations of ten hydrophobic contacts (labeled in Figure 9a) in the last 60 ns MD simulation.

Figure 6a shows all possible hydrophobic and hydrogen-bonding contacts between helices E and G in the bound 14-3-3σ structure identified with LigPlot+ [32]. To further investigate the interactions between helices E and G, comparisons of the populations of ten hydrophobic contacts between apo-14-3-3σ simulations and bound 14-3-3σ during the last 60 ns MD simulation were performed and shown in Figure 6b. As can be seen in Figure 6b, the population of the ten contacts for apo-14-3-3σ is different from that for bound 14-3-3σ. Three hydrophobic contacts with populations higher than 50% are found in bound 14-3-3σ, and no hydrophobic contacts with populations higher than 50% is found in apo-14-3-3σ. This implies that the interaction between helices E and G for bound 14-3-3σ are more stable than for apo-14-3-3σ.

A hydrogen bond between the side chains of Asp126 and Asn175 is observed in the crystallographic structure. In order to evaluate the stability of the hydrogen bond, we investigate the hydrogen bond between E and G in bound 14-3-3σ and apo-14-3-3σ during the MD simulation. For bound 14-3-3σ, two stable hydrogen bonds are formed between the two oxygen atoms (named OD1 and OD2) of Asp 126 side chain and the nitrogen atom of Asn175 side chain with 99.67% and 87.90% occupancy, respectively. However, the two stable hydrogen bonds are not found in apo-14-3-3σ. In bound 14-3-3σ, the side chain of Arg129 forms a stable hydrogen bond with the phosphorylated group. The four residues formed hydrogen bonds with the phosphorylated group and show stable conformation during the whole MD simulation (Table S1). However, a stable hydrogen bond net is found between the side chain of Glu182 and side chain of Arg129 in apo-14-3-3σ. The minimum distances between the side chain of Glu182 and side chain of Arg129 are calculated for every structure during the last 60 ns MD simulation for apo-14-3-3σ. The minimum distances are less than the largest distance 3.5 Å formed, a hydrogen bond with 72.5%. This implies that the conformation of the four residues in the bound 14-3-3σ no longer exists in the apo-14-3-3σ.

### 2.6. The Reason for the Conformational Change

**Figure 7.**
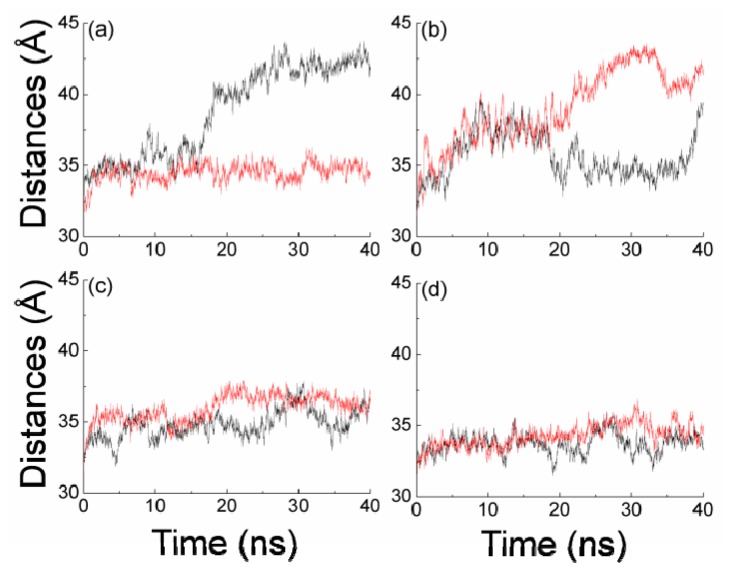
Plots of distances between the mass center of the helices A to D in the two monomers and the mass center of the helices G to I throughout the MD simulations. (**a**) is for nop-14-3-3σ; (**b**) is for apo-14-3-3σ with four residues restrained; (**c**,**d**) are for bound 14-3-3 with Arg56 and Arg129 mutated to alanine residues. The black and red lines are for the monomer A and monomer B, respectively.

The phosphorylated group plays a key role for 14-3-3 protein to identify the target protein. To investigate the role of the phosphorylated group in 14-3-3σ, the 60 ns MD simulation is performed for bound 14-3-3σ without the phosphorylated group (nop-14-3-3σ). The relative position of the helices A to D and G to I and the interactions between the two monomers are stable during the apo-14-3-3σ and bound 14-3-3σ MD simulation process. So it is reasonable to define the state of 14-3-3σ by using the distances between the mass center of helices A to D in the two monomers and the mass center of helices G to I. Figure 7 plots the distances between the mass center of the helices A to D in two monomers and the mass center of the helices G to I tracked through MD simulations. Important information is found in Figure 7a, the monomers A and B are in open and closed states after the first 20 ns MD simulation, respectively. This implies that there may be some other residues playing the same function as the phosphorylated group for 14-3-3σ protein to bind the target proteins.

**Figure 8.**
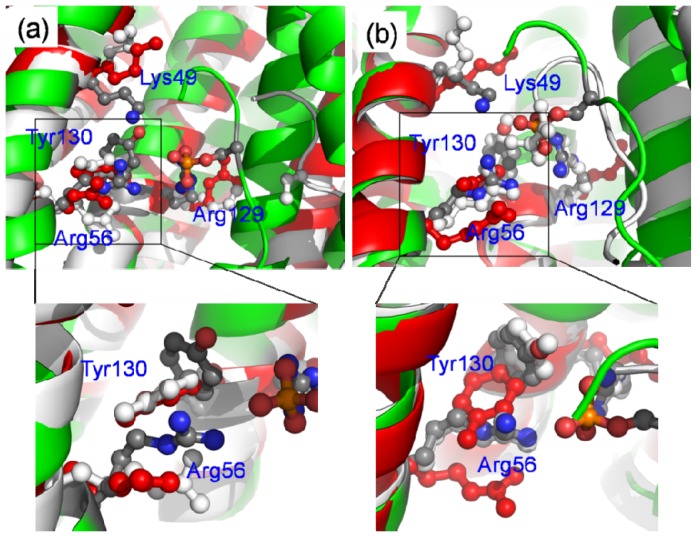
Superimposition of the key residues from the last structure for bound 14-3-3σ, apo-14-3-3σ and nop-14-3-3σ. The proteins are shown in cartoon representation, apo-14-3-3 is shown in green color, bound 14-3-3 in red and nop-14-3-3σ in white. The key residues are shown in ball and stick representation. (**a**) is for monomer A; (**b**) is for monomer B.

Comparisons of the key residues in the last structure of the MD simulation for the three systems (bound 14-3-3σ, apo-14-3-3σ and nop-14-3-3σ) were performed and are shown in Figure 8. It is obvious as seen in Figure 8a that the conformations of the four residues (Lys49, Arg56, Arg129 and Tyr130) in apo-14-3-3σ are different from those in bound 14-3-3σ and same as those in nop-14-3-3σ. The relative position of the peptide in nop-14-3-3σ is different from that in bound 14-3-3σ. The reasons are that the nop-14-3-3σ is in an open state and the bound 14-3-3σ in a closed state. This implies that the phosphorylated group plays an important role in the binding between the 14-3-3σ protein and the target protein. However, as shown in Figure 8b, where different conditions were observed, the conformations of the four residues except Lys49 in bound 14-3-3σ are similar as those in nop-14-3-3σ and different from those in apo-14-3-3σ. The relative position of the peptide in nop-14-3-3σ is similar as that in bound 14-3-3σ except the *C*-terminal of the peptide. The *C*-terminal forms a few hydrogen bonds with the four residues. This indicates that there would be some other residues which play the same role as the phosphorylated residues. This result is in accordance with earlier studies, which show that the 14-3-3 proteins can bind their ligands in a phosphorylation-independent manner [27,33,34].

As shown in Figure 8, the four residues (Lys49, Arg56, Arg129 and Tyr130) would be in close proximity when 14-3-3 protein is in a closed state. Our data suggest that the state of the 14-3-3σ protein is determined by the conformations of the key four residues. In order to test the possibility, another MD simulation was carried out in which the relative position of the four residues in the monomer A are restrained. The distances between the mass center of helices A to D in two monomers and the mass center of helices G to I are plotted in Figure 7b. The conformation of monomer A is in a closed state during the 20 to 38 ns MD simulation times and the monomer B is in an open state. This implies that the conformation of the four residues may play some role for the 14-3-3σ states. In order to evaluate the role of residues Arg56 and Arg129, two additional MD simulations for the bound 14-3-3σ with Arg56 or Arg129 mutated to alanine residues are performed. The distances between the mass center of helices A to D in two monomers and the mass center of helices G to I are plotted in Figure 7c,d. Comparisons between Figures 7c and S2b,c, reveal that the distances are larger in Figure 7 than in Figure S2b and smaller than in Figure S2c. This implies that the residue Arg56 would play some function in maintaining the state of 14-3-3σ protein, as well as Arg129.

## 3. Materials and Methods

### 3.1. System Setups

In this study, the crystal structure of 14-3-3σ determined by Wilker *et al*. was used as the template structure (PDB ID: 1YWT) [12]. Missing loops were obtained from the crystal structure of 14-3-3σ (PDB ID: 3MHR) [5]. MD trajectories were generated for two states. One state was for bound 14-3-3σ, the other one for apo-14-3-3σ. In addition, the bound 14-3-3σ systems were dephosphorylated to get the nop-14-3-3σ systems, restrained four key residues (Lys49, Arg56, Arg129 and Tyr130) and mutated the Arg56 or Arg129 to alanine, respectively. The standard AMBER force field (FF03) [35] was used to describe the protein parameters, the parameters of phosphoserine was designed by Homeye *et al*. [36]. The 14-3-3σ proteins were solvated in a rectangular periodic box of TIP3P [37] water molecules with a margin distance of 12 Å, and the system neutralized by adding an appropriate number of sodions. The apo-14-3-3σ and bound 14-3-3σ systems contain 74,315 and 80,107 atoms, respectively.

### 3.2. Molecular Dynamics Simulation

All the MD simulations are carried out using the AMBER10 package [38]. Periodic boundary conditions and particle mesh Ewald method [39] were employed to treat long-range electrostatic interactions. All the covalent bonds involving hydrogen atoms were constrained by applying the SHAKE algorithm [40]. The integration time step for all MD simulations was set at 2 fs. The nonbonded cutoff was 12 Å. The solvated models were first minimized with the module SANDER in constant volume by 1000 cycles of steepest descent minimization followed by 1000 cycles of conjugated gradient minimization. After energy minimization, applying harmonic restraints with force constants of 2 kcal/(mol·Å^2^) to all solute atoms, canonical ensemble (NVT)-MD was carried out for 70 ps, during which the systems were heated from 0 to 300 K. Subsequent isothermal isobaric ensemble (NPT)-MD is used for 90 ps to adjust the solvent density. Finally, 80 ns isothermal isobaric ensemble (NPT)-MD simulation is applied to apo-14-3-3σ and bound 14-3-3σ simulations without any restraints. The nop-14-3-3σ and the mutated systems are run for 40 ns. The restrained systems were run for 40 ns applying harmonic restraints with force constants of 2 kcal/(mol·Å^2^). The temperature was regulated at 300 K using a Langevin thermostat and the pressure was kept at 1.0 atm using isotropic positional scaling. The intermediate structures were saved at every 1 ps for analysis.

### 3.3. Free Energy Calculations

The free energies for 14-3-3σ proteins were calculated by using MM-GBSA method. All water molecules and counterions were stripped. For every snapshot, the total energy (*G*_total_) was estimated from contribution of gas-phase energies (*H*_gas_) and solvation free energies (*G*_solvation_).

(1) $$G_{\text{total}}=H_{\text{gas}}+G_{\text{solvation}}$$

*H*_gas_ was further divided into a van der Waals (*E*_vdW_) and electrostatic energies (*E*_ele_). These energies were computed using the same parameters set as that used in the MD simulation. And the solvation free energy (*G*_solvation_) was further divided into a polar (*G*_pol_) and a non-polar component (*G*_nonpol_).

(2) $$H_{\text{gas}}=E_{\text{vdW}}+E_{\text{ele}}$$

(3) $$G_{\text{solvation}}=G_{\text{pol}}+G_{\text{nonpol}}$$

The polar contribution (*G*_pol_) was calculated by generalized Born (GB) methods implemented in sander. The non-polar free energy (*G*_nonpol_) is determined using,

(4) $$G_{\text{nonpol}}=\gamma \text{SASA}+\beta$$

where SASA is the solvent-accessible surface area that is determined using the LCPO model [41]. The values γ and β are the empirical constants and are set 0.005 kcal/(mol·Å^2^) and 0, respectively [42].

The conformational entropy contributions were estimated for 300 snapshots evenly from the last 80 ns MD trajectories using normal-mode analysis with AMBER NMODE module [43].

Free energy decomposition in terms of contributions from structural subunits of both binding partners provides insight into the origin of binding on an atomic level. We demonstrated the decomposition on a per-residue basis into contributions from molecular mechanics and solvation energies but not for entropies. The binding interaction of each monomer-residue pair includes four terms: van der Waals contribution (Δ*E*_vdW_), electrostatic contribution (Δ*E*_ele_), polar solvation contribution (Δ*G*_pol_) and non-polar solvation contribution (Δ*G*_nonpol_)

(5) $$\Delta G_{\text{monmer}-\text{residue}}=\Delta E_{\text{vdW}}+\Delta E_{\text{ele}}+\Delta G_{\text{pol}}+\Delta G_{\text{nonpol}}$$

### 3.4. Structural Analysis

Ptraj [44] module of Amber Tools software is used for hydrogen bond analysis, RMSD, RMSF and DSSP. The formation of the hydrogen bond depended on the distance and the orientation criteria as follow: (1) the distance between donor (D) and acceptor (A) atoms is shorter than or equal 3.5 Å; (2) the angle D-H...A is greater or equal to 120°.

## 4. Conclusions

MD simulations were carried out to evaluate the conformational differences between apo-14-3-3σ and bound 14-3-3σ. The apo-14-3-3σ and bound 14-3-3σ were in the same original closed state. However, apo-14-3-3σ was in an open state after the first 20 ns, and bound 14-3-3σ was in a closed state during the whole 80 ns MD simulation. Comparisons of the conformational character between the open state and the closed state were performed. The relative position for the nine helices except E and F was stable in both systems. The interactions between the two monomers were stable for both systems. The conformational change for apo-14-3-3σ from the closed state to the open state mainly causes different interactions between helices E and G. The two monomers of MD simulation for non phosphopeptide are in the closed and open states, respectively. This implies the 14-3-3σ protein may bind to not only phosphopeptide but also non phosphopeptide. The restrained four residues (Lys49, Arg56, Arg129 and Tyr130) MD simulation indicates that the conformation of these four residues play an important role for 14-3-3σ protein in an open or closed state. The four could bind to not only the phosphorylated residues but also other molecules with stronger polarity. These results would be helpful to understand the structure-function relationship of 14-3-3σ protein, and to support drug design of 14-3-3σ protein.

## Supplementary Information



## Figures and Tables

**Table 1.** Free energies for 14-3-3σ protein (kcal/mol).

14-3-3σ	Enthalpic contribution	Entropic contribution	Total energy
Apo-14-3-3σ	−5532.83 ± 43.46	2666.32 ± 6.46	−8199.15
Bound 14-3-3σ	−5520.95 ± 43.62	2658.52 ± 6.90	−8179.47

## References

[1] Aitken A. (2006). 14-3-3 proteins: A historic overview. Semin. Cancer Biol.

[2] Fu H., Subramanian R.R., Masters S.C. (2000). 14-3-3 proteins: Structure, function, and regulation. Annu. Rev. Pharmacol. Toxicol.

[3] Dubois T., Rommel C., Howell S., Steinhussen U., Soneji Y., Morrice N., Moelling K., Aitken A. (1997). 14-3-3 is phosphorylated by casein kinase I on residue 233. Phosphorylation at this site *in vivo* regulates Raf/14-3-3 interaction. J. Biol. Chem.

[4] Powell D.W., Rane M.J., Joughin B.A., Kalmukova R., Hong J.H., Tidor B., Dean W.L., Pierce W.M., Klein J.B., Yaffe M.B. (2003). Proteomic identification of 14-3-3zeta as a mitogen-activated protein kinase-activated protein kinase 2 substrate: role in dimer formation and ligand binding. Mol. Cell. Biol.

[5] Schumacher B., Skwarczynska M., Rose R., Ottmann C. (2010). Structure of a 14-3-3sigma-YAP phosphopeptide complex at 1.15 A resolution. Acta Crystallogr. Sect. F.

[6] Urano T., Saito T., Tsukui T., Fujita M., Hosoi T., Muramatsu M., Ouchi Y., Inoue S. (2002). Efp targets 14-3-3 sigma for proteolysis and promotes breast tumour growth. Nature.

[7] Chan T.A., Hermeking H., Lengauer C., Kinzler K.W., Vogelstein B. (1999). 14-3-3sigma is required to prevent mitotic catastrophe after DNA damage. Nature.

[8] Hermeking H., Benzinger A. (2006). 14-3-3 proteins in cell cycle regulation. Semin. Cancer Biol.

[9] Pozuelo Rubio M., Geraghty K.M., Wong B.H., Wood N.T., Campbell D.G., Morrice N., Mackintosh C. (2004). 14-3-3-affinity purification of over 200 human phosphoproteins reveals new links to regulation of cellular metabolism, proliferation and trafficking. Biochem. J.

[10] Yang X., Lee W.H., Sobott F., Papagrigoriou E., Robinson C.V., Grossmann J.G., Sundstrom M., Doyle D.A., Elkins J.M. (2006). Structural basis for protein-protein interactions in the 14-3-3 protein family. Proc. Natl. Acad. Sci. USA.

[11] Liu D., Bienkowska J., Petosa C., Collier R.J., Fu H., Liddington R. (1995). Crystal structure of the zeta isoform of the 14-3-3 protein. Nature.

[12] Wilker E.W., Grant R.A., Artim S.C., Yaffe M.B. (2005). A structural basis for 14-3-3sigma functional specificity. J. Biol. Chem.

[13] Gardino A.K., Smerdon S.J., Yaffe M.B. (2006). Structural determinants of 14-3-3 binding specificities and regulation of subcellular localization of 14-3-3-ligand complexes: A comparison of the X-ray crystal structures of all human 14-3-3 isoforms. Semin. Cancer Biol.

[14] Yaffe M.B., Rittinger K., Volinia S., Caron P.R., Aitken A., Leffers H., Gamblin S.J., Smerdon S.J., Cantley L.C. (1997). The structural basis for 14-3-3:phosphopeptide binding specificity. Cell.

[15] Rittinger K., Budman J., Xu J., Volinia S., Cantley L.C., Smerdon S.J., Gamblin S.J., Yaffe M.B. (1999). Structural analysis of 14-3-3 phosphopeptide complexes identifies a dual role for the nuclear export signal of 14-3-3 in ligand binding. Mol. Cell.

[16] Coblitz B., Wu M., Shikano S., Li M. (2006). *C*-terminal binding: An expanded repertoire and function of 14-3-3 proteins. FEBS Lett.

[17] Bustos D.M. (2012). The role of protein disorder in the 14-3-3 interaction network. Mol. Biosyst.

[18] Milroy L.G., Brunsveld L., Ottmann C. (2013). Stabilization and inhibition of protein-protein interactions: The 14-3-3 case study. ACS Chem. Biol.

[19] Liu J.Y., Li Z., Li H., Zhang J.T. (2011). Critical residue that promotes protein dimerization: A story of partially exposed Phe25 in 14-3-3sigma. J. Chem. Inf. Model.

[20] Li Z., Peng H., Qin L., Qi J., Zuo X., Liu J.Y., Zhang J.T. (2013). Determinants of 14-3-3sigma protein dimerization and function in drug and radiation resistance. J. Biol. Chem.

[21] Benzinger A., Popowicz G.M., Joy J.K., Majumdar S., Holak T.A., Hermeking H. (2005). The crystal structure of the non-liganded 14-3-3sigma protein: Insights into determinants of isoform specific ligand binding and dimerization. Cell Res.

[22] Scott K.A., Randles L.G., Moran S.J., Daggett V., Clarke J. (2006). The folding pathway of spectrin R17 from experiment and simulation: Using experimentally validated MD simulations to characterize States hinted at by experiment. J. Mol. Biol.

[23] Ji C.G., Zhang J.Z. (2011). Understanding the molecular mechanism of enzyme dynamics of ribonuclease A through protonation/deprotonation of HIS48. J. Am. Chem. Soc.

[24] Chen H.F., Luo R. (2007). Binding induced folding in p53-MDM2 complex. J. Am. Chem. Soc.

[25] Hu G., Chen L.Y., Wang J. (2012). Insights into the mechanisms of the selectivity filter of *Escherichia coli* aquaporin Z. J. Mol. Model.

[26] Verma S., Singh A., Mishra A. (2013). The effect of fulvic acid on pre- and postaggregation state of Abeta17–42. Molecular dynamics simulation studies. BBA-Proteins Proteomics.

[27] Obsil T., Obsilova V. (2011). Structural basis of 14-3-3 protein functions. Semin. Cell Dev. Biol.

[28] Joosten R.P., te Beek T.A., Krieger E., Hekkelman M.L., Hooft R.W., Schneider R., Sander C., Vriend G. (2011). A series of PDB related databases for everyday needs. Nucleic Acids Res.

[29] Hu G., Zhang Q., Chen L.Y. (2011). Insights into scFv: Drug binding using the molecular dynamics simulation and free energy calculation. J. Mol. Model.

[30] Gohlke H., Kiel C., Case D.A. (2003). Insights into protein-protein binding by binding free energy calculation and free energy decomposition for the Ras-Raf and Ras-RalGDS complexes. J. Mol. Biol.

[31] Chen J., Zhang D., Zhang Y., Li G. (2012). Computational studies of difference in binding modes of peptide and non-peptide inhibitors to MDM2/MDMX based on molecular dynamics simulations. Int. J. Mol. Sci.

[32] Laskowski R.A., Swindells M.B. (2011). LigPlot+: Multiple ligand-protein interaction diagrams for drug discovery. J. Chem. Inf. Model.

[33] Fu H., Coburn J., Collier R.J. (1993). The eukaryotic host factor that activates exoenzyme S of *Pseudomonas aeruginosa* is a member of the 14-3-3 protein family. Proc. Natl. Acad. Sci. USA.

[34] Henriksson M.L., Troller U., Hallberg B. (2000). 14-3-3 proteins are required for the inhibition of Ras by exoenzyme S. Biochem. J.

[35] Yong D., Chun W., Shibasish C., Mathew C.L., Guoming X., Wei Z., Rong Y., Piotr C., Ray L., Taisung L. (2003). A point-charge force field for molecular mechanics simulations of proteins based on condensed-phase quantum mechanical calculations. J. Comput. Chem.

[36] Homeyer N., Horn A.H., Lanig H., Sticht H. (2006). AMBER force-field parameters for phosphorylated amino acids in different protonation states: Phosphoserine, phosphothreonine, phosphotyrosine, and phosphohistidine. J. Mol. Model.

[37] Jorgensen W.L., Chandrasekhar J., Madura J.D., Impey R.W., Klein M.L. (1983). Comparison of simple potential functions for simulating liquid water. J. Comput. Phys.

[38] Case D.A., Darden T.A., Cheatham T.E., Simmerling C.L., Wang J., Duke R.E., Luo R., Crowley M., Walker R.C., Zhang W. (2008). *AMBER*, version 10.

[39] Darden T., York D., Pedersen L. (1993). Particle mesh ewald and Log(*N*) method for ewald sums in large systems. J. Comput. Phys.

[40] Ryckaert J.P., Ciccotti G., Berendsen H.J.C. (1977). Numerical-integration of cartesian equations of motion of a system with constraints-molecular-dynamics of *N*-alkanes. J. Comput. Phys.

[41] Weiser J., Shenkin P.S., Still W.C. (1999). Approximate atomic surfaces from linear combinations of pairwise overlaps (LCPO). J. Comput. Chem.

[42] Onufriev A., Bashford D., Case D.A. (2004). Exploring protein native states and large-scale conformational changes with a modified generalized born model. Proteins.

[43] Case D.A. (1994). Normal mode analysis of protein dynamics. Curr. Opin. Struct. Biol.

[44] Case D.A., Cheatham T.E., Darden T., Gohlke H., Luo R., Merz K.M., Onufriev A., Simmerling C., Wang B., Woods R.J. (2005). The Amber biomolecular simulation programs. J. Comput. Chem.

