# Supplementary material for: Insight into Conformational Change for 14-3-3σ Protein by Molecular Dynamics Simulation
Source: Int J Mol Sci. 2014 Feb 18;15(2):2794–810. doi: 10.3390/ijms15022794 (PMC3958882; doi:10.3390/ijms15022794)
Supplement: Supplementary file 1 [file ijms-15-02794-s001.pdf]

Supplementary Information

Table S1. Hydrogen bonds formed between the phosphorylated residues and 14-3-3σ protein.

Donor	Acceptor	%Occupancy	Distance (Å)	Angle (°)
Sep-O1P	Arg56-HH12-NH1	70.57	3.13 ± 0.25	141.42 ± 13.17
Sep-O1P	Arg56-HH22-NH2	93.80	2.86 ± 0.16	160.09 ± 11.37
Sep-O1P	Arg129-HH12-NH1	99.92	2.78 ± 0.11	164.72 ± 7.93
Sep-O1P	Arg129-HH22-NH2	21.35	3.37 ± 0.11	129.25 ± 4.98
Sep-O2P	Lys49-HZ1-NZ	11.52	3.19 ± 0.21	140.32 ± 13.13
Sep-O2P	Lys49-HZ2-NZ	12.10	3.19 ± 0.21	139.95 ± 12.93
Sep-O2P	Lys49-HZ3-NZ	10.40	3.20 ± 0.22	140.32 ± 13.62
Sep-O2P	Arg129-HH12-NH1	36.25	3.35 ± 0.12	131.27 ± 5.77
Sep-O2P	Arg129-HH22-NH2	100.00	2.78 ± 0.10	166.27 ± 7.12
Sep-O2P	Tyr130-HH-OH	99.98	2.68 ± 0.12	166.08 ± 7.28
Sep-O3P	Lys49-HZ1-NZ	29.50	2.80 ± 0.14	149.35 ± 13.42
Sep-O3P	Lys49-HZ2-NZ	32.70	2.80 ± 0.13	149.85 ± 13.52
Sep-O3P	Lys49-HZ3-NZ	29.23	2.80 ± 0.13	149.89 ± 13.89
Sep-O3P	Arg56-HH12-NH1	89.45	2.96 ± 0.19	156.27 ± 12.81
Sep-O3P	Arg56-HH22-NH2	5.35	3.42 ± 0.07	130.61 ± 5.57
Ter-OXT	Arg56-HH12-NH1	98.35	2.81 ± 0.12	156.62 ± 11.78
Ter-OXT	Arg56-HH22-NH2	41.70	3.20 ± 0.21	135.19 ± 8.74
Ter-O	Arg56-HH12-NH1	49.70	3.21 ± 0.19	139.52 ± 9.94
Ter-O	Arg56-HH22-NH2	94.85	2.92 ± 0.17	159.57 ± 10.33
Ter-O	Arg129-HH12-NH1	95.30	2.88 ± 0.16	153.88 ± 10.93

Figure S1. The apo-14-3-3σ (colored in cyan) structure superimposed with the bond 14-3-3σ (colored in red) structure. The protein is shown in cartoon representation, and the phosphopeptide is shown in ball and stick representation. The nine helices in monomer A are labeled.

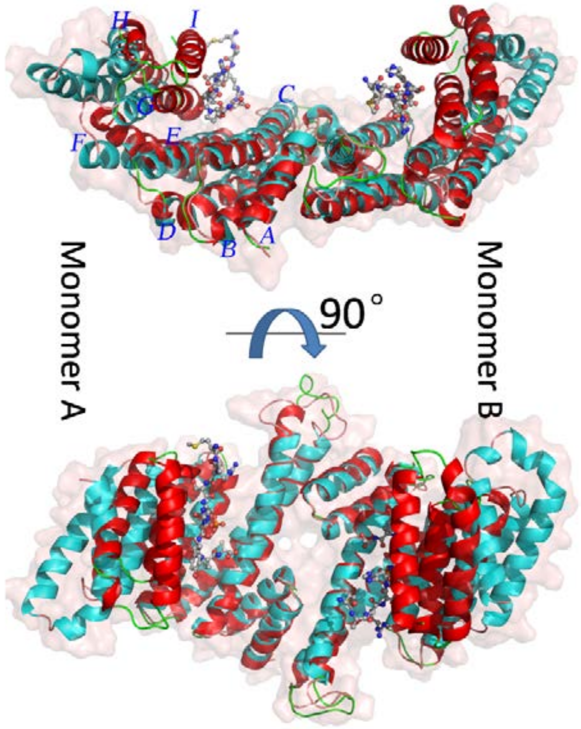

Figure S2. (**a**) The distances between the mass center of the helices G, H and I for both monomers as a function of MD simulation time. The slopes of the linear regression lines are 7.05 × 10^{-7} and −8.52 × 10^{-6} Å/ps; (**b**) The distances between the mass center of the helices A to D in the both monomers and the mass center of the helices G, H and I as a function of MD simulation time for bound 14-3-3σ; as well as (**c**) for apo-14-3-3σ.

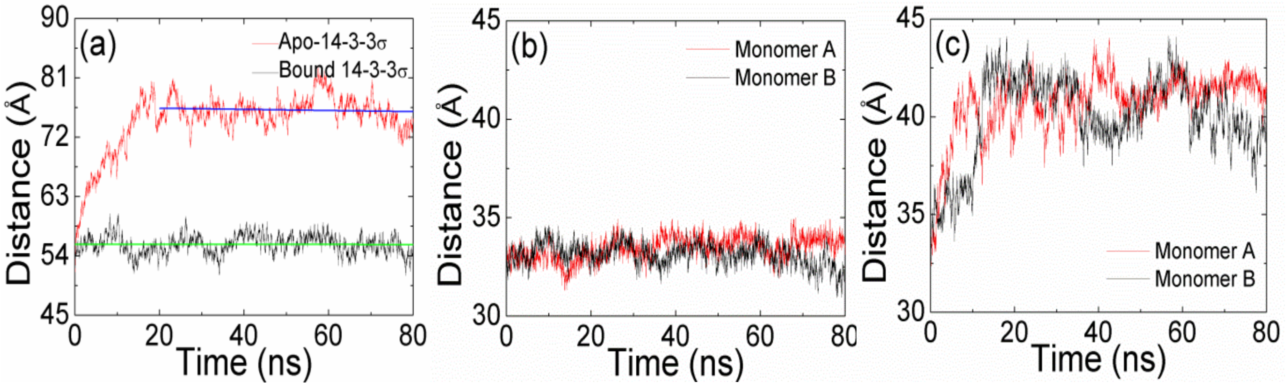

Figure S3. Evolution of DSSP as a function of the MD simulation time for (**a**) apo-14-3-3σ and (**b**) bound 14-3-3σ, respectively. (0 for none, 1 for parallel beta-sheet, 2 for anti-parallel beta-sheet, 3 for 3–10 helix, 4 for alpha helix, 5 for Pi (3–14) helix and 6 for turn).

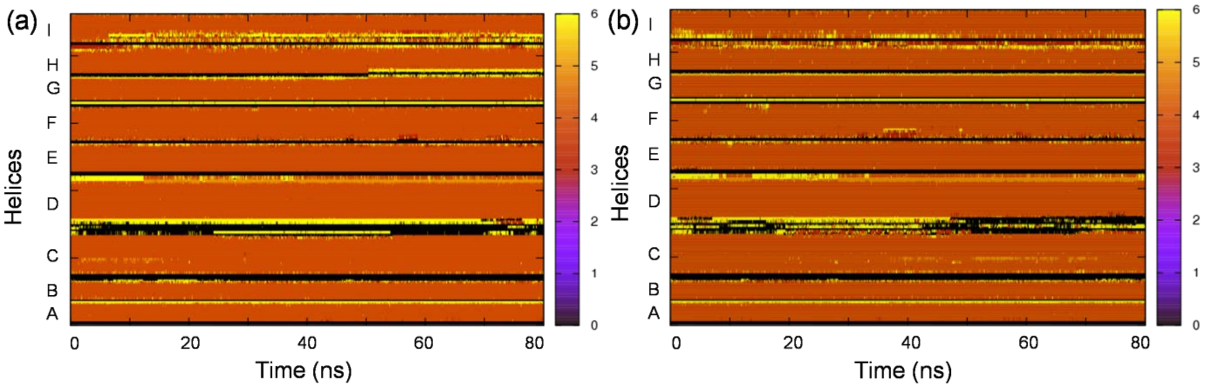

Figure S4. RMSDs of backbone atoms as a function of the MD simulation time. (**a**) is for the helices A, B, C and D; and (**b**) is for helices G, H and I.

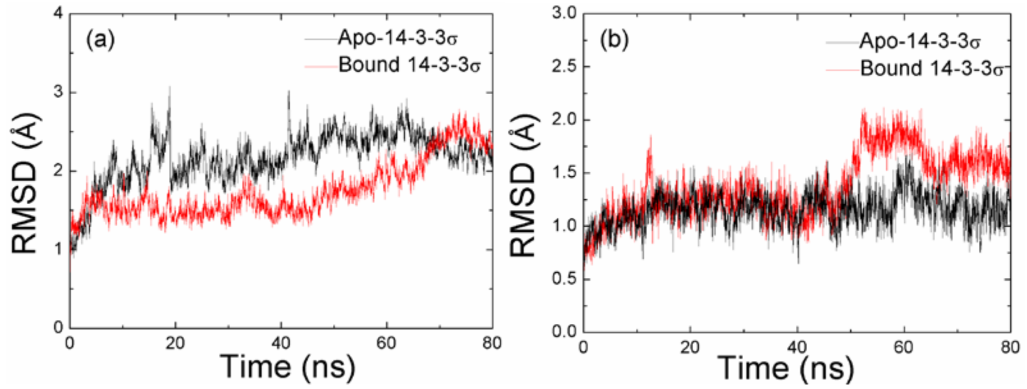

Figure S5. (**a**) RMSDs of backbone atoms of the helices E, F and G as a function of the MD simulation time; (**b**) Distances tracked through MD simulation between the mass center of helices E and G.

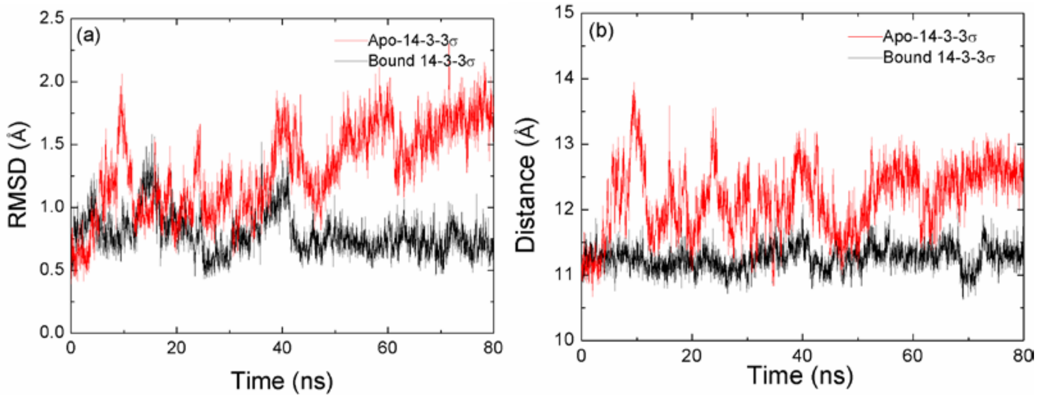

© 2014 by the authors; licensee MDPI, Basel, Switzerland. This article is an open access article distributed under the terms and conditions of the Creative Commons Attribution license (http://creativecommons.org/licenses/by/3.0/).
